# Supplementary material for: Delivery system can vary ventilatory parameters across multiple patients from a single source of mechanical ventilation
Source: PLoS One. 2020 Dec 10;15(12):e0243601. doi: 10.1371/journal.pone.0243601 (PMC7728450; doi:10.1371/journal.pone.0243601)
Supplement: S1 Raw data — (ZIP) [file pone.0243601.s001.zip › 1025-027 Surgery Data.pdf]

Sedative \ Anesthesia \ Analgesia Administration

Study Number 1025-027

Animal Number 1001 **Group** 1 **Sex** F **Body Weight**[a] 43.50 (kg) g

Sedative/Anesthetic/Analgesic Administration ***

Product Name	Interval of Administration^	Sterile Water Added (mL)	Dose (mg/kg)	Concentration (mg/mL)	Calculated Dose (mL)	Actual Dose[α] (mL)	Route	Technician*	Recorder* ƶ	Time
Glycopyrrolate	PD	NA	0.01	0.2	**2.2**		IM	E	D	0857
Euthanasia Solution	PS	NA	NA	NA	**10.0**		IV	E	B	1627

Induction ***

Product Name	Interval of Administration^	Amount of Sterile Water Added (mL)	Dose (mg/kg)	Concentration (mg/mL)	Calculated Dose (mL)	Actual Dose[α] (mL)	Route	Technician*	Recorder* ƶ	Time
Ketamine	PD	NA	15	100	**6.5**		IM	E	D	0857
Xylazine	PD	NA	0.22	100	**0.10**		IM	E	D	0857

Maintenance ***

Product Name	Dose**	Route	Technician*	Recorder* ƶ	Start Time	End Time
Sevoflurane	To Effect	INH	FE	F	0908	1003
NA	NA	NA	NA	NA	NA	NA

Fluids ***

Product Name	Dose (mL/hour)	Route	Technician*	Recorder* ƶ
Sodium Chloride 0.9%	**90**	IV	F	

IV catheter placed by*: DfE **Shaved by*:** NA **Eye lube placed by*:** I **Intubated by*:** FE

Calculations conducted by:	[a]Calculations Verified by:	*** Drug Table Modifications Approved by:
JRB 25Jun2020	[illegible] 26Jun2020	

[a]Body weights measured on 25-Jun-2020

Transcribed body weights confirmed by personnel verifying calculations.

***Function completed by** (initials and date) ƶ: **A:** ______ **B:** [illegible] 26Jun2020 **C:** ______ **D:** [illegible] 26Jun2020 **E:** [illegible] 26Jun2020 **F:** [illegible] 26Jun2020 **G:** ______ **H:** ______

α - Needed if different from calculated ƶ - The recorder's letter, initials, and date are required if a technician other than the recorder performs the function.

TD - Transdermal INH - Inhalation INF - Infused into incisions PRA - Rectal Administration ** - Delivered in oxygen

^If applicable, drug given: PD - Prior to Induction, PI-prior to incision, PS- post-surgery, IO- intra-operative, PR- prior to scan, PT- post-scan

Sedative \ Anesthesia \ Analgesia Administration

Study Number 1025-027

Animal Number 2001 **Group** 2 **Sex** M **Body Weight**[a] 86.00 (kg) g

Sedative/Anesthetic/Analgesic Administration ***

Product Name	Interval of Administration^	Sterile Water Added (mL)	Dose (mg/kg)	Concentration (mg/mL)	Calculated Dose (mL)	Actual Dose[α] (mL)	Route	Technician*	Recorder* z	Time
Atropine Sulfate	PD	NA	0.05	0.54	8.0		IM	D		0752
Acetaminophen	IO	NA	per vet	per vet	650mg		PRA	G	F	1356
Acetaminophen	IO	NA	Per vet	per vet	650mg		PRA	G		1013
Acetaminophen	IO	NA	Per vet	Per vet	650mg		PRA	G		1117
Euthanasia Solution	PS	NA	NA	NA	15.0		IV	B	C	1623

Induction ***

Product Name	Interval of Administration^	Amount of Sterile Water Added (mL)	Dose (mg/kg)	Concentration (mg/mL)	Calculated Dose (mL)	Actual Dose[α] (mL)	Route	Technician*	Recorder* z	Time
Ketamine	PD	NA	22	100	18.9		IM	D	G	~~0817~~
Xylazine	PD	NA	2	100	1.7		IM	D	G	0817

Maintenance ***

Product Name	Dose**	Route	Technician*	Recorder* z	Start Time	End Time
Sevoflurane	To Effect	INH	E		0825	09:04
NA	NA	NA	NA	NA	NA	NA

Fluids ***

Product Name	Dose (mL/hour)	Route	Technician*	Recorder* z
①②③④ Sodium Chloride 0.9%	170	IV	E	

IV catheter placed by*: D, E **Shaved by*:** NA **Eye lube placed by*:** E **Intubated by*:** D, E

Calculations conducted by:	[a]Calculations Verified by:	*** Drug Table Modifications Approved by:
JRB 25Jun2020	[illegible] 26Jun2020	SMT 26 JUN 2020

[a]Body weights measured on 25-Jun-2020

Transcribed body weights confirmed by personnel verifying calculations.

***Function completed by** (initials and date) z: **A:** ______ **B:** ______ **C:** CMD 26Jun2020 **D:** DMA 26Jun2020 **E:** [illegible] 26Jun2020 **F:** KAM 26Jun2020 **G:** JLG 26Jun2020 **H:** ______

α - Needed if different from calculated z - The recorder's letter, initials, and date are required if a technician other than the recorder performs the function.

TD - Transdermal INH - Inhalation INF - Infused into incisions PRA - Rectal Administration ** - Delivered in oxygen

^If applicable, drug given: PD - Prior to Induction, PI-prior to incision, PS- post-surgery, IO- intra-operative, PR- prior to scan, PT- post-scan

① fluids decreased to 100ml/hr @ 1000. JLG 26Jun2020.
② fluids decreased to 60ml/hr @ 1144. JLG 26Jun2020.
③ fluids increased to 80ml/hr @ 1326. JLG 26JUN2020.
④ Fluids increased to 100ml/hr at 1502. KAM 26Jun2020

Anesthesia Monitoring Record w/ Pressure

Study Number: 1025-027

Animal Number 1001 **Group** 1 **Sex** F

Δ - MM / CRT Check	Body Temp °F	Heart Rate (BPM)	Expired CO_2	Respiration/ Min	Pulse O_2 %	Mean Arterial Pressure	Mean Venous Pressure	Oxygen (L/Min)	Tech*	Time
Δ	102.8	103	22	75	96	NA	NA	2.0	F	0930
Δ	100.9	119	68	13	97	NA	NA	2.0	D,E	0948
Δ	100.9	113	63	13	97	NA	NA	2.0	D,E	0953
Δ	100.9	109	61	13	97	NA	NA	2.0	D,E	0958
Δ	100.9	106	55	14	97	NA	NA	2.0	D,E	1003
Δ	100.8	109	53	14	97	NA	NA	2.0	D,E	1008
Δ	100.8	110	52	14	98	NA	NA	2.0	D,E	1013
Δ	100.8	112	51	14	97	NA	NA	2.0	D,E	1018
Δ	100.8	112	52	14	97	NA	NA	2.0	D,E	1023
Δ	100.9	111	52	14	98	NA	NA	2.0	D,E	1028
Δ	101.9	111	51	14	97	NA	NA	2.0	D,E	1033
Δ	101.3	111	50	14	98	① ~~NA~~ 82	NA	2.0	D,E	1038
Δ	101.5	110	50	14	98	85	NA	2.0	D,E	1043
Δ	101.1	109	49	14	97	84	NA	2.0	D,E	1048
Δ	101.5	109	49	12	97	86	NA	2.0	D,E	1053
Δ	101.7	99	49	14	97	84	NA	2.0	D,E	1058
Δ	100.8	107	49	14	97	80	NA	2.0	D,E	1103
Δ	101.3	106	49	14	97	77	NA	2.0	E	1110
Δ	101.8	105	49	14	96	75	NA	2.0	E	1115
Δ	102.0	103	50	14	97	70	NA	2.0	E	1120
Δ	102.4	103	50	14	97	70	NA	2.0	E	1125
Δ	102.4	102	50	14	97	68	NA	2.0	E	1130
Δ	102.4	102	49	14	97	65	NA	2.0	E	1135
Δ	102.6	101	50	14	97	62	NA	2.0	D,E	1140
Δ	② ~~101~~ 102.6	100	49	14	97	61	NA	2.0	D,E	1145
Δ	102.6	100	49	14	97	59	NA	2.0	D,E	1150
Δ	102.7	100	50	14	97	97	NA	2.0	D,E	1155
Δ	102.6	100	50	14	97	52	NA	2.0	D,E	1210
Δ	102.6	100	50	14	97	49	NA	2.0	D,E	1225
Δ	102.6	101	50	14	97	43	NA	2.0	D,E	1240
Δ	102.6	102	46	14	97	49	NA	2.0	D,E	1255
Δ	102.4	100	45	14	97	45	NA	2.0	D,E	1310
Δ	101.8	99	45	14	98	45	NA	2.0	D,E	1325

Δ - Mucous Membranes (MM)/Tissue Checked (color and/or capillary refill time{CRT}); BPM - Beats per minute
L/Min - Liters per minute [s] **- Product:** Sevoflurane

* - A letter indicates the function was completed by (Initials/Date):

A - ______ B - ______ C - ______
D - DMA 26Jun2020 E - [signature] 26Jun2020 F - KAM 26Jun2020
G - ______ H - ______ I - ______

① Correction of value DMA 26Jun2020
② documented in wrong spot DMA 26Jun2020

Anesthesia Monitoring Record w/ Pressure

Study Number: 1025-027

Animal Number 1001 **Group** 1 **Sex** F

Δ - MM / CRT Check	Body Temp °F	Heart Rate (BPM)	Expired CO_2	Respiration/ Min	Pulse O_2 %	Mean Arterial Pressure	(1) Mean Venous Pressure	Oxygen (L/Min)	Tech*	Time
Δ	102.2	98	45	14	97	43	(4) 17/3	2.0	D, E	1330
Δ	102.2	98	44	14	97	42	NA	2.0	D, E	1335
Δ	102.2	97	43	14	97	41	NA	2.0	D, E	1340
Δ	102.2	97	44	14	97	40	23/10 (3)	2.0	A	1345
Δ	102.2	97	43	14	97	40	24/09 (3)	2.0	A	1350
Δ	102.2	97	43	14	97	39	22/09 (2)	2.0	D, E	1355
Δ	102.2	96	43	14	96	40	NA	2.0	D, E	1400
Δ	102.4	96	44	13	97	37	17/3	2.0	D, E	1405
Δ	102.2	98	53	16	95	48	11/3	2.0	D, E	1410
Δ	102.0	99	60	16	94	36	14/2	2.0	D, E	1415
Δ	102.0	100	45	17	97	36	17/2	2.0	D, E	1420
Δ	102.0	100	51	17	96	42	16/1	2.0	D, E	1425
Δ	102.0	99	44	17	97	37	16/1	2.0	D, E	1430
Δ	101.8	95	43	17	97	38	16/1	2.0	D, E	1435
Δ	101.8	93	45	16	97	41	10/1	2.0	D, E	1440
Δ	101.8	95	48	16	96	36	15/1	2.0	D, E	1445
Δ	101.8	96	49	17	96	37	15/1	2.0	D, E	1450
Δ	101.8	97	54	17	96	38	12/1	2.0	D, E	1455
Δ	101.7	100	55	17	95	33	15/1	2.0	D, E	1500
Δ	101.7	101	51	17	95	37	15/1	2.0	D, E	1505
Δ	101.7	102	53	17	95	34	15/1	2.0	D, E	1510
Δ	101.7	100	48	17	95	37	15/1	2.0	D, E	1515
Δ	101.7	97	46	17	94	40	15/1	2.0	D, E	1520
Δ	101.5	94	46	17	93	38	15/1	2.0	D, E	1525
Δ	101.5	91	46	17	94	37	15/1	2.0	D, E	1530
Δ	101.5	89	45	17	94	35	15/1	2.0	D, E	1535
Δ	101.5	88	45	17	94	35	15/1	2.0	B	1540
Δ	101.5	87	44	17	94	33	15/1	2.0	B	1545
Δ	101.5	86	45	17	94	34	15/1	2.0	B	1550
Δ	101.5	86	44	17	95	33	15/1	2.0	B	1555
Δ	101.5	85	44	17	94	34	15/1	2.0	B	1600
Δ	101.5	85	44	17	95	32	15/1	2.0	B	1605
Δ	101.5	85	44	17	94	33	15/1	2.0	B	1610

Δ - Mucous Membranes (MM)/Tissue Checked (color and/or capillary refill time{CRT}); BPM - Beats per minute

L/Min - Liters per minute [6] - Product: Sevoflurane

* - A letter indicates the function was completed by (Initials/Date):

A - ggo 26 June 2020 B - [illegible] 26JUN2020 C -

D - DMA 26 Jun 2020 E - [illegible] 26 Jun 2020 F -

G - H - I -

(4) ~~(2)~~ (1) clarification should read vent Pressure DMA 26Jun2020

(2) on balloon DMA 26Jun2020 (4) placement error ggo 26 June 2020

(3) on balloon ggo 26 June 2020

Anesthesia Monitoring Record w/ Pressure

Study Number: 1025-027

Animal Number 1001 **Group** 1 **Sex** F

	Δ - MM / CRT Check	Body Temp °F	Heart Rate (BPM)	Expired CO_2	Respiration/ Min	Pulse O_2 %	Mean Arterial Pressure	Mean Venous Pressure	Oxygen (L/Min)	Tech*	Time
	Δ	101.5	85	45	17	94	33	13/1	2.0	B	1615
	Δ	101.5	85	42	17	94	31	15/1	2.0	B	1620
	Δ	101.5	85	42	17	94	32	16/1	2.0	B	1625
①	NA	101.5	0	14	17	0	NA	2/0	2.0	B	1629

Δ - Mucous Membranes (MM)/Tissue Checked (color and/or capillary refill time{CRT}); BPM - Beats per minute

L/Min - Liters per minute [6] - Product: Sevoflurane

* - A letter indicates the function was completed by (Initials/Date):

A - ________ B - BRy 26Jun2020 C - ________

D - ________ E - ________ F - ________

G - ________ H - ________ I - ________

① Animal Euthanised at 1627 BRy 26Jun2020

Anesthesia Monitoring Record w/ Pressure

Study Number: 1025-027

Animal Number 2001 **Group** 2 **Sex** M

Δ - MM / CRT Check	Body Temp °F	Heart Rate (BPM)	Expired CO_2	Respiration/ Min	Pulse O_2 %	② Mean Arterial Pressure	③ Mean Venous Pressure	Oxygen (L/Min)	Tech*	Time
Δ	101.6	121	48	30	99	NA	NA	2.0	D,E	0843
Δ	100.6	105	49	14	97	NA	NA	2.0	B	09:05
Δ	100.2	109	43	14	98	NA	NA	2.0	G	0916
Δ	100.2	110	42	14	97	NA	NA	2.0	G	0920
Δ	100.4	109	41	14	98	NA	NA	2.0	G	0925
Δ	100.6	106	40	14	99	NA	NA	2.0	G	0930
Δ	NA	104	39	14	97	NA	112	2.0	G	0935
Δ	101.5	105	39	14	99	NA	119	2.0	G	0940
Δ	101.7	102	38	14	98	NA	125	2.0	G	0945
Δ	101.7	99	38	14	99	NA	128	2.0	G	0950
Δ	101.8	99	37	14	98	NA	124	2.0	G	0955
Δ	102.8	96	37	14	99	NA	132	2.0	G	1000
Δ	102.6	99	37	14	99	NA	135	2.0	G	1010
Δ	102.7	91	36	14	99	NA	137	2.0	G	1016
Δ	102.7	92	37	14	98	NA	138	2.0	G	1020
Δ	102.9	88	37	14	96	NA	130	2.0	G	1026
Δ	103.1	87	37	14	98	NA	140	2.0	F	1031
Δ	103.1	86	36	14	98	NA	140	2.0	F	1036
Δ	103.3	84	36	14	97	NA	138	2.0	F	1041
Δ	103.3	84	41	16	98	NA	136	① ~~35%~~ 2.5	F	1047
Δ	103.3	84	40	16	98	NA	137	2.75	F	1052
Δ	103.1	81	41	15	98	NA	135	0.0	F	1057
Δ	103.3	87	41	16	99	NA	131	5.0	F	1103
Δ	103.5	88	41	16	98	11	128	5.0	F	1108
Δ	103.5	88	44	16	97	12	123	5.0	F	1113
Δ	102.9	88	43	16	98	10	125	5.0	G	1121
Δ	103.6	88	45	17	97	10	122	5.0	G	1129
Δ	103.6	88	48	~~98~~ ④	NA	11	121	5.0	G	1140
Δ	103.6	84	52	10	96	7	96	5.0	G	1150
Δ	103.8	79	41	14	95	6	124	5.0	G	1155
Δ	103.6	80	39	14	94	⑤ ~~0.0~~ NA	121	2.0	F	1200
Δ	103.5	86	37	14	90	NA	121	2.0	F	1208
Δ	103.5	78	37	14	94	NA	120	2.0	F	1213

Δ - Mucous Membranes (MM)/Tissue Checked (color and/or capillary refill time{CRT}); BPM - Beats per minute
L/Min - Liters per minute [6] - Product: Sevoflurane

* - A letter indicates the function was completed by (Initials/Date):

A - ______ B - JRB 26Jun2020 C - ______
D - DMA 26Jun2020 E - ______ F - KAM 26 JUN 2020
G - JLG 26JUN2020 H - ______ I - ______

① written in error. KAM 26JUN2020
② Clarification. Should read "Mean Vent Pressure". KAM 26JUN2020
③ Clarification. Should read "Mean Arterial pressure". JLG 26JUN2020.
④ incorrect. Should read "NA", JLG 26JUN2020
⑤ Correction. KAM ~~JUN2020~~ ⑥
⑥ Correction of date. Should read "26JUN2020". KAM 29JUN2020

Anesthesia Monitoring Record w/ Pressure

Study Number: 1025-027

Animal Number 2001 **Group** 2 **Sex** M

Δ - MM / CRT Check	Body Temp °F	Heart Rate (BPM)	Expired CO_2	Respiration/ Min	Pulse O_2 %	Mean Arterial Pressure	(2) Mean Venous Pressure	(1) Oxygen (L/Min)	Tech*	Time
Δ	103.3	78	39	16	97	119	14/2	36.7	F	1221
Δ	103.3	78	40	15	96	118	14/2	33.0	F	1226
Δ	103.3	77	41	16	96	117	14/3	33.7	F	1231
Δ	103.3	77	42	16	96	114	14/3	33.6	F	1236
Δ	103.3	76	43	16	96	112	14/3	33.6	F	1241
Δ	103.3	75	48	16	96	106	12/3	46.5	F	1247
Δ	103.3	74	46	16	97	109	12/3	51.6	F	1251
Δ	103.5	73	46	16	97	107	13/2	52.3	F	1257
Δ	103.5	72	53	16	97	105	10/2	32.7	F	1301
Δ	103.5	72	46	16	97	105	13/3	56.5	F	1307
Δ	103.5	72	45	16	96	102	13/3	56.3	F	1312
Δ	103.5	71	45	16	97	99	14/3	56.0	F	1317
Δ	103.5	69	57	16	95	86	11/3	65.7	F	1322
Δ	103.6	70	47	16	96	98	14/2	56.5	F	1330
Δ	103.6	69	44	16	98	97	14/3	63.5	F	1335
Δ	103.8	69	42	16	96	98	14/3	66.4	F	1340
Δ	104.0	68	49	18	97	95	12/3	66.8	F	1345
Δ	104	67	41	16	97	94	15/3	63.0	G	1350
Δ	104.0	67	39	16	97	86	15/2	62.6	F	1355
Δ	104.0	67	38	16	95	94	14/3	62.7	F	1400
Δ	104.0	67	43	16	97	89	14/3	33.6	F	1405
Δ	104.2	64	54	16	95	84	12/3	64.6	F	1410
Δ	104.2	63	59	16	96	81	14/4	61.3	F	1415
Δ	104.2	65	42	17	97	87	17/4	70.8	F	1420
Δ	104.2	63	50	17	98	62	19/7	66.2	F	1425
Δ	104.2	64	40	17	98	78	19/5	69.1	F	1430
Δ	104.4	63	40	17	98	72	18/5	68.1	F	1437
Δ	104.4	61	42	17	99	57	18/5	67.8	F	1444
Δ	104.4	61	43	17	99	56	18/5	67.3	F	1450
Δ	104.4	62	43	17	99	67	18/5	70.5	F	1455
Δ	104.4	61	45	17	98	57	18/5	67.3	F	1500
Δ	104.5	60	36	17	99	60	20/5	55.4	F	1507
Δ	104.4	59	42	17	99	64	18/5	67.1	F	1512

Δ - Mucous Membranes (MM)/Tissue Checked (color and/or capillary refill time{CRT}); BPM - Beats per minute

L/Min - Liters per minute **6 - Product:** **Sevoflurane**

* - A letter indicates the function was completed by (Initials/Date):

A - ______ B - ______ C - ______

D - ______ E - ______ F - KAM 26JUN2020

G - JLG 26JUN2020 H - ______ I - ______

(1) Clarification. Unit of measurement should read "FiO_2". KAM 26JUN2020

(2) clarification. Should read "vent pressure". KAM 26JUN2020

Anesthesia Monitoring Record w/ Pressure

Study Number: 1025-027

Animal Number 2001 **Group** 2 **Sex** M

Δ - MM / CRT Check	Body Temp °F	Heart Rate (BPM)	Expired CO_2	Respiration/ Min	Pulse O_2 %	Mean Arterial Pressure	② Mean Venous Pressure	① Oxygen (L/Min)	Tech*	Time
Δ	104.4	57	41	17	99	60	24/-1	67.0	F	1517
Δ	104.4	57	40	17	99	58	19/5	67.9	F	1522
Δ	104.5	60	41	17	98	62	18/5	67.1	G	1525
Δ	104.5	62	41	17	99	64	18/5	67.9	G	1531
Δ	104.5	60	41	17	99	60	20/5	67.3	G	1535
Δ	104.5	58	41	17	99	58	18/5	67.2	G	1541
Δ	104.5	61	42	16	98	65	19/5	67.2	C	1546
Δ	104.5	61	41	17	98	61	19/5	67.0	C	1551
Δ	104.7	60	41	17	98	58	16/5	67.7	C	1556
Δ	104.7	60	41	17	98	57	23/2	66.4	G	1601
Δ	104.7	61	41	17	98	57	19/5	67.5	G	1605
Δ	104.7	60	41	17	98	55	19/5	67.2	C	1610
Δ	104.7	61	~~60~~ ③	17	97	59	20/3	66.6	C	1615
Δ	104.7	52	50	16	98	31	24/10	63.7	C	1620
④ Δ	104.7	0	17	17	NA	15	24/12	66.1	C	1626

Δ - Mucous Membranes (MM)/Tissue Checked (color and/or capillary refill time{CRT}); BPM - Beats per minute

L/Min - Liters per minute ^s^ - Product: Sevoflurane

④ time of euthanasia. CMD 26JUN2020

* - A letter indicates the function was completed by (Initials/Date):

A - 　　B - 　　C - CMD 26JUN2020

D - 　　E - 　　F - KAM 26JUN2020

G - JLG 26JUN2020 　　H - 　　I -

① Clarification. Unit of measurement should read "FiO_2". KAM 26JUN2020

② Clarification. Should read "vent pressure". KAM 26JUN2020

③ incorrect should read "39". CMD 26JUN2020

Study #: 1025-027

Animal Number: 1001 Group: 1 Sex: F Body Weight*: 43.5 kg

Initials†	Procedures
JRB	The animal was placed on the surgical table in dorsal recumbency and draped for surgery.
JRB	A small incision was made in the inguinal region(s).
JRB	A cervical incision was made on the ventral neck to access the appropriate vessel(s).
	**Vessel Use:**
JRB	**RIGHT Femoral - Artery** The vessel was located, isolated and instrumented.<br>A 5 F ☑ Sheath was placed in the vessel for (*check all that apply*):<br>☐ Blood Sampling ☐ Systemic BP monitoring ☐ Drug Administration
JRB	**RIGHT Femoral - Vein** The vessel was located, isolated and instrumented.<br>A 5 F ☑ Sheath was placed in the vessel for (*check all that apply*):<br>☐ Blood Sampling ☐ Systemic BP monitoring ☐ Drug Administration
NA	**LEFT Femoral - Artery** The vessel was located, isolated and instrumented.<br>A _____ F ☐ Sheath was placed in the vessel for (*check all that apply*):<br>☐ Blood Sampling ☐ Systemic BP monitoring ☐ Drug Administration
NA	**LEFT Femoral - Vein** The vessel was located, isolated and instrumented.<br>A _____ F ☐ Sheath was placed in the vessel for (*check all that apply*):<br>☐ Blood Sampling ☐ Systemic BP monitoring ☐ Drug Administration
JRB	**RIGHT Jugular - Vein** The vessel was located, isolated and instrumented.<br>A 8 F ☑ Sheath was placed in the vessel for (*check all that apply*):<br>☑ Blood Sampling ☑ Systemic BP monitoring ☐ Drug Administration
JRB	① ~~**LEFT Jugular - Vein**~~ The vessel was located, isolated and instrumented.<br>A 8 F ☑ Sheath was placed in the vessel for (*check all that apply*):<br>☐ Blood Sampling ☑ Systemic BP monitoring ☐ Drug Administration
JRB	iStat (CG8+) samples were collected and run throughout the procedure per protocol.
JRB	Datalogger was started at least 15 minutes prior to study start.

Comments and/or footnotes (as applicable): ① Should read "Right Carotid" JRB 26Jun2020

Lung tissue harvested and placed into 10% formalin

[illegible] 26 Jun 2020

	Initials and Date
Recorder(s)	JRB 26Jun2020
Surgeon	[illegible] 26 Jun 2020
Surgical Assistant	NA

Study #: 1025-027

Animal Number: 2001 Group: 2 Sex: M Body Weight*: 86.0 kg

Initials†	Procedures
JRB	The animal was placed on the surgical table in dorsal recumbency and draped for surgery.
JRB	A small incision was made in the inguinal region(s).
JRB	A cervical incision was made on the ventral neck to access the appropriate vessel(s).
	**Vessel Use:**
JRB	**RIGHT Femoral - Artery** The vessel was located, isolated and instrumented. A 8 F ☑ **Sheath** was placed in the vessel for (*check all that apply*): ☐ **Blood Sampling** ☐ **Systemic BP monitoring** ☐ **Drug Administration**
JRB	**RIGHT Femoral - Vein** The vessel was located, isolated and instrumented. A 5 F ☑ **Sheath** was placed in the vessel for (*check all that apply*): ☐ **Blood Sampling** ☐ **Systemic BP monitoring** ☐ **Drug Administration**
NA	**LEFT Femoral - Artery** The vessel was located, isolated and instrumented. A ____ F ☐ **Sheath** was placed in the vessel for (*check all that apply*): ☐ **Blood Sampling** ☐ **Systemic BP monitoring** ☐ **Drug Administration**
NA	**LEFT Femoral - Vein** The vessel was located, isolated and instrumented. A ____ F ☐ **Sheath** was placed in the vessel for (*check all that apply*): ☐ **Blood Sampling** ☐ **Systemic BP monitoring** ☐ **Drug Administration**
JRB	**RIGHT Jugular - Vein** The vessel was located, isolated and instrumented. A 5 F ☑ **Sheath** was placed in the vessel for (*check all that apply*): ☐ **Blood Sampling** ☐ **Systemic BP monitoring** ☐ **Drug Administration**
JRB	① ~~**LEFT Jugular - Vein**~~ The vessel was located, isolated and instrumented. A 8 F ☑ **Sheath** was placed in the vessel for (*check all that apply*): ☐ **Blood Sampling** ☑ **Systemic BP monitoring** ☐ **Drug Administration**
JJO	iStat (CG8+) samples were collected and run throughout the procedure per protocol.
JRB	Datalogger was started at least 15 minutes prior to study start.

Comments and/or footnotes (as applicable): ① Should read "Right Carotid" JRB 26Jun2020

Lung tissue harvested and placed in 10% formalin

JJO 26 June 2020

	Initials and Date
Recorder(s)	JRB 26Jun2020
Surgeon	JJO 26 June 2020
Surgical Assistant	NA

Maintenance Anesthesia, Drugs and Emergency Drug Record, Study #: 1025-027

Animal #: 2001 Group Number: 2 Sex: M Weight: 86.0 kg

Bolus Anesthesia and Emergency Drugs

Diazepam Bolus		Propofol Bolus		Emergency Drug given IV	Amount (mL)	Time
Sheep 0.1mg/kg IV (0.87mL)	Pig 0.2mg/kg IV (3.4mL)	Sheep 3mg/kg IV (13.1mL)	Pig 3mg/kg IV (25.8mL)			
NA		NA	✓		10	1144
NA		NA		50% Dextrose①	10	1508
NA		NA				
NA		NA				
NA		NA				
NA		NA				
NA		NA				
NA		NA				
NA		NA				
NA		NA				
NA		NA				
NA		NA				
NA		NA				
NA		NA				
NA		NA				
NA		NA				
NA		NA				
NA		NA				
NA		NA				
NA		NA				
NA		NA				
NA		NA				

Comments and/or Footnotes:

① given with 50mL Saline IV JJO 26 Jun 2020

	Initials and Date
Recorder	JJO 26 June 2020
Drug Administrator	JJO 26 June 2020
Surgeon or Veterinarian	JJO 26 June 2020

Study #: 1025-027

Animal Number: 1001 Group: 1 Sex: F

ISTAT Information

Time Taken	iSTAT (CG8+) X	[redacted]	Time Taken	iSTAT (CG8+) X
* 10:02	X			
1203	X			
1400	X			
1415	X			
1430	X			
1445	X			
1500 ①	X			
1515	X			
1522	X			
1530	X			
1545	X			
1600	X			
1615	X			
1623	X final			

* Baseline; X - Completed

Comments and/or footnotes (as applicable):
① Cartridge error [illegible] 26 June 2020

	Initials and Date
Recorder (s)	JRB 26Jun2020 [illegible] 26 June 2020

Study #: 1025-027

Animal Number: 2001 **Group:** 2 **Sex:** M

ISTAT Information

Time Taken	iSTAT (CG8+) X	[redacted]	Time Taken	iSTAT (CG8+) X
* 09:18	X			
1112	X			
1129	X			
1145	X			
1227	X			
1244	X			
1315	X			
1331	X			
1344	X			
1359	X			
1415	X			
1430	X			
1445	X			
1500	X			
1515	X			
1530	X			
1545	X			
1600	X			
1615	X			
1622	X final			

* Baseline; X - Completed

Comments and/or footnotes (as applicable):

	Initials and Date
Recorder (s)	JRB 26Jun2020 [illegible] 26 June 2020

Record of Anesthesia Infusion
Study #: 1025-027

Animal #: 1001 Group#/Sex: 1F Body Weight:* 43.5 kg Pump ID: INFP 340

KXT CRI Infusion Start Time / Time of Rate Changes	KXT CRI Infusion Rate mL/hour	KXT CRI Infusion Rate Changes (Time)	KXT CRI Infusion Rate mL/hour	KXT CRI Infusion Rate Changes (Time)	KXT CRI Infusion Rate mL/hour
Start: 09:43	87				
1232	65				
1629	0				

KXT CRI – Ketamine Xylazine Telazol Continuous Rate Infusion

Ketamine (2.0mg/ml solution) (4mg/kg/hour) = __174__mg/hr = ___87___mL/hour †

Xylazine (0.1mg/ml solution) (0.2mg/kg/hour) = __8.7__mg/hr = ___87___mL/ hour †

Telazol (3.0mg/ml solution) (6mg/kg/hour) = __261__mg/hr = ___87___mL/hour †

Calculated By: JRB 25Jun2020 **Verified By:** [illegible] 26Jun2020

†The above doses are intended as a guide and may be adjusted to ensure an adequate depth of anesthesia.
*Transcribed from Provantis body weight recorded on 25Jun2020

Comments and/or Footnotes (*as applicable*):

	Initials and Date
Recorder	JRB 26Jun2020 [illegible] 26June2020
Drug Administrator	JRB 26Jun2020

Record of Anesthesia Infusion

Study #: 1025-027

Animal #: 2001 Group#/Sex: 2M Body Weight:* 86.0 kg Pump ID: INFP 2510

KXT CRI Infusion Start Time / Time of Rate Changes	KXT CRI Infusion Rate mL/hour	KXT CRI Infusion Rate Changes (Time)	KXT CRI Infusion Rate mL/hour	KXT CRI Infusion Rate Changes (Time)	KXT CRI Infusion Rate mL/hour
Start: 08:52	172				
1139	260				
1326	215				
1624	0				

KXT CRI – Ketamine Xylazine Telazol Continuous Rate Infusion

Ketamine (2.0mg/ml solution) (4mg/kg/hour) = 344 mg/hr = 172 mL/hour †

Xylazine (2.0mg/ml solution) (4mg/kg/hour) = 344 mg/hr = 172 mL/ hour †

Telazol (3.0mg/ml solution) (6mg/kg/hour) = 516 mg/hr = 172 mL/hour †

Calculated By: JRB 25Jun2020 **Verified By:** [illegible] 26Jun2020

†The above doses are intended as a guide and may be adjusted to ensure an adequate depth of anesthesia.
*Transcribed from Provantis body weight recorded on 25Jun2020

Comments and/or Footnotes (*as applicable*):

	Initials and Date
Recorder	JRB 26Jun2020 JLG 26Jun2020
Drug Administrator	JRB 26Jun2020 [illegible] 26Jun2020

CRL Study # 1025-027

Animal ID: 2001 Group#: 2 Sex: M

Testing Conditions and Parameters	[redacted]								
Time:	12:20 (1)	12:30 (2)	12:35 (2)	12:40 (2)	12:45 (3)	12:50 (3)	12:55 (3)	1301 (4)	13:05
VTE (ml)	615	585	570	570	595	480	495	365	520
P.Max (cmH_2O)	20	23	22	22	24	21	21	18	24
PEEP (cm H20)	4	4	4	4	3	3	3	3	3
MV (L)	9.8	9.0	9.1	9.1	9.2	7.6	7.8	5.8	8.3
Freq. (per minute)	16	16	16	16	16	16	16	16	16
FiO_2 (%)	36	33.8	33.6	33.7	57.7	50.4	51.8	32.8	56.0
SPO2 %	97	96	96	96	97	97	94	97	97
Resp. Pressure (mmHg)	14/2 5	14/3 6	13/3 6	13/3 4	13/2 5	12/3 6	13/3 5	10/2 5	13/2 6
End Time	12:22	12:31	12:35	12:40	12:48	12:51	1255	1301	13:05
Acclimation Time (min):	10 B	5 B	5 B	5 B	5 B	5 B	5 D	6 D	4 B

*using an ambu-bag ᵅ Skin flap (pig only)

A:	C:	Comments:
B: JRB 26Jun2020	D: [illegible] 26Jun2020	

(1) Baseline JRB 26Jun2020
(2) Stand by mode JRB 26Jun2020
(3) Switched at 12:45; leveled for 2 min prior to parameters, Main + Balloon JRB 26Jun2020
(4) rest period JRB 26Jun2020

CRL Study # 1025-027

Animal ID: 2001 Group#: 2 Sex: M

Testing Conditions and Parameters	[redacted]								
Time:	13:10 R	13:15 R	13:20 P	13:25 P (5)	13:30 P (5)	13:35 P (5)	13:40 P (5)	13:45 P (5)	13:50 P (5)
VTE (ml)	520	525	527	510	520	575	595	631	560
P.Max (cmH_2O)	25	25	25	24	25	30	34	39	39
PEEP (cm H20)	3	3	3	3	3	3	3	3	3
MV (L)	8.3	8.3	8.3	8.1	8.3	9.2	9.5	8.9	9.8
Freq. (per minute)	16	16	16	16	16	16	16	16	16
FiO_2 (%)	56.2	56.9	57.1	55.1	56.7	63.5	66.7	63.3	64.6
SPO2 %	96	96	96	97	96	98	96	96	97
Resp. Pressure (mmHg)	14/3 6	14/3 6	13/2 6	(4) 16/3 7<br>(5) 13/3 5	(4) 17/3 7<br>(5) 13/3 7	(4) 19/2 6<br>(5) 14/3 6	(4) 22/2 7<br>(5) 14/3 6	(4) 22/24 10 18<br>(5) 15/2 6	(4) 23/9 12<br>(5) 15/3 7
End Time	13:10	13:15	13:20	13:25	(3) 13:33<br>13:30	(3) 13:38<br>13:35	13:40	13:45	13:51
Acclimation Time (min):	5 B	5 B	5 B	5 B	5 B	5 B	5 B	5 B	5 B

*using an ambu-bag ᵅSkin flap (pig only)

(3) Pressure to balloon increased JRB26Jun2020 (6) PEEP Booster JRB26Jun2020

A:	C:	Comments: R-Rest; P-Piggyback + Balloon; C-Coughing;
B: JRB26Jun2020	D:	D-Dyssynchrony; OC-Open Circuit; OCC-Occluded Circuit;

IL-In Line PEEP Regulator (4) Balloon JRB26Jun2020

(1) Sheep JRB26Jun2020 (5) Pig JRB26Jun2020

CRL Study # 1025-027

Animal ID: 2001 Group#: 2 Sex: M

Testing Conditions and Parameters	[redacted]								
Time:	13:55 P,PB	14:00 P,PB	14:05 P,PB	14:10 PS,PB ①	14:15 PS,PB	14:20 PS,PB	1425 PS,PB	14:30 PS,PB	14:35 PS,PB
VTE (ml)	1050	1050	570	620	580	640	525	580	585
P.Max (cmH₂O)	34	33	22	25	31	32	29	32	32
PEEP (cm H20)	3	3	4	3	2	3	1	2	2
MV (L)	10.4	10.4	9.0	9.6	10.6	10.9	9.0	9.8	9.9
Freq. (per minute)	16	16	16	16	16	17	17	17	17
FiO₂ (%)	62.6	62.4	33.6	62.8	70.8	71.8	67.4	69.2	69.5
SPO2 %	96	96	97	98 Sw / 97 S	97 Sw / 97 S	97 Sw / 97 S	98 Sw / 96 S	98 Sw / 97 S	98 Sw / 96 S
Resp. Pressure (mmHg)	22/9 12S / 14/3 7Sw	22/9 12S / 15/2 6Sw	16/2 6S / 14/3 6Sw	14/3 6S / 14/2 6Sw	19/3 8S / 18/4 8Sw	16/3 8S / 18/4 9Sw	19/5 9Sw / 16/1 5S	16/1 7S / 18/5 10Sw	16/1 6S / 18/5 10Sw
End Time	13:55	14:00	14:05	14:12	14:15	14:20	1425	14:30	14:35
Acclimation Time (min):	5 B	5 B	5 B	5 B	5 B	5 B	5D	5B	5 B

*using an ambu-bag ᵅSkin flap (pig only) ① PB added at 14:13 JRB 26Jun2020

A:	C:	Comments: R-Rest; P-Piggyback + Balloon; C-Coughing; D-Dyssynchrony;
B: JRB 26Jun2020	D: [illegible] 26Jun2020	OC-Open Circuit; OCC-Occluded Circuit; IL-In Line PEEP reg.;

PB-PEEP Booster; S-Sheep; Sw-Pig; PS-Piggyback w/sheep;

CRL Stud' [illegible] 1025-027

Animal ID: 2001 Group#: 2 Sex: M

Testing Conditions and Parameters	[redacted]								
Time:	14:40 PS PB	14:45 PS PB	14:50 PS PB α	14:55 PS,α PB	15:00 PS PB	16:05 PS,α PB	15:10 PS PB	15:15 PS PB	1520 PS PB
VTE (ml)	550	565	560	465	550	750	565	560	570
P.Max (cmH_2O)	32	33	31	33	30	32	33	32	32
PEEP (cm H20)	1	2	2	1	2	2	2	2	2
MV (L)	8.5	9.6	9.4	9.2	9.1	12.7	9.6	9.4	9.6
Freq. (per minute)	17	17	17	17	17	17	17	17	17
FiO_2 (%)	59.4	67.2	67.1	71.2	66.9	54.2	68.0	67.2	67.3
SPO2 %	98 SW / 96 S	99 SW / 96 S	99 SW / 96 S	98 SW / 96 S	99 SW / 96 S	99 SW / 91 S	98 SW / 95 S	99 SW / 95 S	98 SW / 93 S
Resp. Pressure (mmHg)	10/1 4 S / 18/7 9 SW	15/1 7 S / 18/5 10 SW	15/1 7 S / 18/5 9 SW	10/1 4 S / 17/5 8 SW	15/1 5 S / 18/5 9 SW	22/1 7 S / 20/5 10 SW	15/1 6 S / 18/5 9 SW	21/2 10 SW / 15/1 7 S	18/5 8 SW / 15/1 5 S
End Time	14:40	14:45	14:50	14:55	15:00 ① ②	15:06	15:10	15:15	1520
Acclimation Time (min):	5 B	5 B	5 B	5B	5B	5B	5B	5 D	5 D

*using an ambu-bag α Skin flap (pig only)

A:	C:	Comments: SW = Pig S = Sheep PS = Piggyback w/Sheep
B: JRB 26Jun2020	D: [illegible] 26Jun2020	PB = Peep Booster

① 15:03 Sheep disconnect from piggyback system; JRB 26Jun2020
② Sheep expired CO_2 increased to 63 JRB 26Jun2020

CRL Study # 1025-027

Animal ID: 2001 Group#: 2 Sex: M

Testing Conditions and Parameters	[redacted]								
Time:	15:25	1530	1535	1540	1545	1550	1555	1600	1605
VTE (ml)	575	580	560	565	565	560	565	555	550
P.Max (cmH_2O)	31	32	31	31	31	32	31	31	31
PEEP (cm H20)	2	2	1	1	1	2	2	2	2
MV (L)	9.5	9.8	9.5	9.6	9.6	9.4	9.5	9.4	9.4
Freq. (per minute)	17	17	17	17	17	17	17	17	17
FiO_2 (%)	67.2	67.4	67.0	67.4	67.0	66.8	66.3	66.6	67.0
SPO2 %	98 Sw<br>93 S	98 Sw<br>94 S	99 Sw<br>94 S	99 Sw<br>94 S	98 Sw<br>94 S	99 Sw<br>94 S	98 Sw<br>95 S	98 Sw<br>94 S	98 Sw<br>95 S
Resp. Pressure (mmHg)	18/5 9 Sw<br>15/1.5 S	18/5 9 Sw<br>15/1.6 S	18/3 9 Sw<br>15/1.6 S	18/5 9 Sw<br>15/1.6 Sw	18/5 10 Sw<br>15/1.6 S	19/5 10 Sw<br>15/1.6 S	19/5 10 Sw<br>15/1.6 S	19/5 10 Sw<br>15/1.5 S	19/5 10 Sw<br>16/1.6 S
End Time	15:25	1530	1535	1540	1545	1550	1555	1600	1605
Acclimation Time (min):	5 D	5 D	5 D	5 D	5 D	5 D	5 D	5 D	5 D

*using an ambu-bag [α]Skin flap (pig only)

A:	C:	Comments: S= Sheep Sw = Pig
B:	D: [signature] 26Jun2020	

CRL Stud[illegible] 1025-027

Animal ID: 2001 Group#: 2 Sex: M

Testing Conditions and Parameters	[redacted]								
Time:	1610	1615	1620	1625					
VTE (ml)	550	625	325	430					
P.Max (cmH_2O)	32	44	37	40					
PEEP (cm H20)	2	1	1	1					
MV (L)	9.2	6.9	5.5	7.2					
Freq. (per minute)	17	17	17	17					
FiO_2 (%)	66.4	66.2	63.4	66.1					
SPO2 %	98 SW<br>94 S	98 SW<br>95 S	98 SW<br>95 S	NA SW<br>95 S					
Resp. Pressure (mmHg)	19/6 10 SW<br>15/1 7 S	26/12/5 SW<br>12/1 5 S	25/10 14 SW<br>16/1 6 S	16/1 7 SW<br>① ~~[illegible]~~ S					
End Time	1610	1615	1620	1625					
Acclimation Time (min):	5 D	5 D	5 D	5 D					

*using an ambu-bag [α]Skin flap (pig only)

A:	C:	Comments: S = Sheep SW = Pig
B:	D: [initials] 26 Jun 2020	

① Correction [initials] 26 Jun 2020
