# Supplementary material for: Delivery system can vary ventilatory parameters across multiple patients from a single source of mechanical ventilation
Source: PLoS One. 2020 Dec 10;15(12):e0243601. doi: 10.1371/journal.pone.0243601 (PMC7728450; doi:10.1371/journal.pone.0243601)
Supplement: Attachment — Submitted filename: Reviewer Response Letter.docx [file pone.0243601.s002.docx]

Delivery system can vary ventilation parameters across multiple patients from a single source of mechanical ventilation

**Response to Reviewers**
11/17/2020
Muhammad Adrish
Academic Editor
PLOS ONE

Dear Dr. Adrish and reviewers;

We greatly appreciate the thoughtful reviews regarding our paper on a novel ventilator-splitting system to address the Coronavirus Pandemic, and would like to thank you for the time reviewing our study. We, too, believe this will be a valuable contribution to the literature and may be particularly useful over the coming months as the pandemic appears to be re-gaining momentum. We have addressed the reviews and critical points as follows:

1. Please ensure that your manuscript meets PLOS ONE's style requirements, including those for file naming.
   a. We have edited the submission to include a separate title page and reformatted the headings within the main body
2. To comply with PLOS ONE submissions requirements, please provide methods of sacrifice in the Methods section of your manuscript.
   a. The methods of sacrifice were a combination of sodium pentobarbital and bilateral pneumothoraxes for both animal studies. This has been added into the methods section [Line 164].
3. Please clarify the relationship between the authors and the approval by the Charles River IACUC.
   a. The authors have no relationship with Charles River. Charles River is an independent animal facility which remained operational during the COVID pandemic while we were unable to complete the 2-animal study internally.
4. Thank you for including your ethics statement:  "University of Michigan Protocol: PRO00009358 Charles River Mattawan IACUC 1025-027".   Please amend your current ethics statement to confirm that your named ethics committee specifically approved this study. Once you have amended this/these statement(s) in the Methods section of the manuscript, please add the same text to the “Ethics Statement” field of the submission form (via “Edit Submission”).
   a. Thank you, we have updated the statement as follows:
   b. University of Michigan Protocol: PRO00009358
      Charles River Mattawan IACUC 1025-027
      The University of Michigan single-animal and Charles River dual-animal experiments were both approved by the respective animal use committees for COVID-19 Research
   c. We also included the following in the methods: “All animal studies were carried out in strict compliance with the Guide for the Care and Use of Laboratory Animals of the National Institutes of Health. The protocol was reviewed by the University of Michigan University Committee on Use and Care of Animals (UCUCA) for the single pig feasibility study, and the Charles River Animal Use Committee for the combined pig and sheep study. Both studies were approved by the respective animal use committees”
5. Thank you for stating the following in the Competing Interests section: [I have read the journal's policy and the authors of this manuscript have the following competing interests: KVK, GEG and DAZ are founders of MakeMedical which is the legal manufacturer of this device (provided at cost).]. Please confirm that this does not alter your adherence to all PLOS ONE policies on sharing data and materials, by including the following statement: "This does not alter our adherence to  PLOS ONE policies on sharing data and materials.”
   a. Thank you for highlighting this. Although we are the legal manufacturers, it does NOT alter our adherence to PLOS ONE policies. Please add the following into our submission:
   b. “I have read the journal's policy and the authors of this manuscript have the following competing interests: KVK, GEG and DAZ are founders of MakeMedical LLC, which is the legal manufacturer of this device (provided at cost). This does not alter our adherence to PLOS ONE policies on sharing data and materials.”
6. Reviewer #1: The authors have done a remarkable work on the concept, which has potential for attenuating ventilator shortages. They have also addressed the limitation of the system which, as intriguing as it sounds, can be also very challenging especially using in high risk population
   a. We thank the reviewer for their thoughtful response and appreciate their comments. We agree this system addresses many of the limitations and concerns regarding ventilator sharing, and believe it is superior to a simple splitting system.
7. Reviewer #2: Comments to the Author
   The authors had developed the new novel delivery system to allow individualized peak inspiratory pressure settings and PEEP using a pressure regulatory valve. The system demonstrated the ability to provide ventilation across clinically relevant scenarios including circuit occlusion, unmatched physiology, and a surgical procedure, while allowing significantly different pressures to be safely delivered to each animal for individualized support.
   Major comments
   - This individual ventilation system had improved the disadvantage and concern of previous non-individualized ventilations system. This study has clinical significance because it aims to prevent medical collapse due to the spread of COVID-19 infection.
   However, I think that you should reveal how to prevent the risk of aerosol infection that may occur during suction treatment in this paper.
   a. We thank Reviewer 2 for their thoughtful comments regarding this system’s capabilities to expand ventilator support with an individualized ventilation system. With traditional vent-splitting, aerosolization is a major concern as single-room isolation is not feasible. At the endotracheal tube connection, the circuit is attached in the same fashion as traditional ventilation tubing. Thus, the system allows for a closed-line suction system to be attached to each patient’s endotracheal tube individually. These closed-line systems would dramatically reduce aerosolization as the circuit remains closed at all times, and would have no significant effect on ventilation.
   b. We have added the following to the paper to address this concern (Line 213). “Since each circuit is connected to the endotracheal tube in a similar fashion to standard ventilation, a closed-line suction system can be sued in each patient to limit aerosolization.”

Thank you so much for consideration of our manuscript in PLOS ONE. We greatly appreciate the time and effort in reviewing our manuscript and have addressed the reviewers concerns. Please let us know if there is anything else that should need to be addressed.

Sincerely,

Kyle K. VanKoevering, MD
